# A study of tumour growth based on stoichiometric principles: a continuous model and its discrete analogue

**DOI:** 10.1080/17513758.2014.913718

**Published:** 2014-05-09

**Authors:** M. Saleem, Tanuja Agrawal, Afzal Anees

**Affiliations:** ^a^ Department of Applied Mathematics, Z.H. College of Engineering and Technology, A.M.U., Aligarh202002, India; ^b^ Department of Surgery, J.N. Medical College, A.M.U., Aligarh202002, India

**Keywords:** predator–prey interactions, ecological stoichiometry, tumour growth, cancer modelling, 92B05, 93D20, 92C50

## Abstract

In this paper, we consider a continuous mathematically tractable model and its discrete analogue for the tumour growth. The model formulation is based on stoichiometric principles considering tumour-immune cell interactions in potassium (K ^+^)-limited environment. Our both continuous and discrete models illustrate ‘cancer immunoediting’ as a dynamic process having all three phases namely elimination, equilibrium and escape. The stoichiometric principles introduced into the model allow us to study its dynamics with the variation in the total potassium in the surrounding of the tumour region. It is found that an increase in the total potassium may help the patient fight the disease for a longer period of time. This result seems to be in line with the protective role of the potassium against the risk of pancreatic cancer as has been reported by Bravi *et al*. [*Dietary intake of selected micronutrients and risk of pancreatic cancer: An Italian case-control study*, Ann. Oncol. 22 (2011), pp. 202–206].

## 1. Introduction

The evidence accumulated in the past decade indicates that the immune system can recognize and eliminate malignant tumours in a process termed ‘cancer immunosurveillance’ [7,22,34]. The work from many laboratories has validated the concept of cancer immunosurveillance, demonstrating that immune system can indeed protect mice from outgrowth of tumours [11,15,30,32,33,39]. The current view on the host immune system is conflicted by evidence for antitumour effects as well as evidence for tumour-favouring actions [39–41]. ‘Cancer immunoediting’ is the term used in the literature for this dual action of the host immune system [39].

The immune system mostly consists of white blood cells, especially T lymphocytes (usually CD8^+^ and CD4^+^ T cells along with their characteristically produced cytokine IFN-γ), natural killer cells and macrophages. The lymphocytes are used to detect any foreign or non-self cells in the body known as antigens. It has been shown experimentally that these immune cells can lyse tumour cells very effectively [7,26]. Tumour associated macrophages or myeloid-derived suppressive cells, CD4^+^ Foxp3^+^ Treg cells and Th17 cells and their associated cytokines Il-6, TNF, IL-1β, IL-23 and TGF-β are generally recognized as dominant tumour-promoting forces [41].

Evidence indicates that a healthy immune system is necessary for control of malignant disease. One of the common factors that have been associated with pronounced abnormalities in the immune system is poor nutrition [23,25]. The convincing evidence exists that individuals who have been on immune-suppressive medications for longer periods of time, or have autoimmune disease or chronic infection (such as AIDS) are particularly at risk of malignancy [24,25]. The chronic inflammation, previous viral infections such as EBV, Hepatitis B and C, herpes virus or HIV are also significant factors that hamper proper functioning of immune system and lead to development of various cancers [12,24,27,31]. Failure of intact immune responses, such as immunosurveillance or immunoediting, has also been associated with evasion or immune-suppression activities of cancer [28,29]. Tumours often avoid detection by Killer T cells by having a reduced number of MHC class I molecules on their surface [10]. Some tumours release products, such as cytokine TGF-β, which suppress the activity of macrophages and lymphocytes [10].

In a recently reported study, Bravi *et al*. [2] considered the role of 15 selected vitamins and carotenoids and 6 minerals including potassium in the protection against pancreatic cancer. Analysing separate role of different minerals, they found significant inverse trends in pancreatic cancer risk for increasing intake of potassium. Prior to Bravi *et al*. ’s [2] work, two studies of Jansson [13,14] regarding the colorectal cancer risk in the USA also hinted at the possible role of electrolytes sodium and potassium in cancer etiology. In its simpler form Jansson studies attempted to suggest that intracellular and dietary potassium (K ^+^) protects against cancer and intracellular and dietary sodium (Na) increases the risk of cancer. It can be mentioned here that all studies such as mentioned above have been of the suggestive nature for the positive role of electrolyte potassium against the risk of cancer. Unfortunately there are neither direct cell-biology-based investigations nor biological data available in the literature that support or negate the role of potassium against cancer risk.

We focus in this paper on the suggestive prediction of the above studies especially the work of Bravi *et al*. [2] regarding the positive role of potassium against the cancer risk. To this end, we formulate a tractable mathematical model based on the principles of stoichiometry to represent interactions of cancer and immune cells in (K ^+^)-limited environment using the structure of the Kuang–Huisman–Elser (KHE) model [16]. We modify this model and incorporate in it the coercing of the surrounding immune cells by cancer cells and a medical treatment strategy that may help add immune cells in the body by a constant rate.

We structure the paper as follows. The continuous model formulation is given in Section 2. The results for boundedness of the continuous model solution and local stability of its equilibriums are given in Section 3. The discrete analogue of the continuous model and the local stability results of its equilibriums are discussed in Section 4. Numerically drawn bifurcation diagrams illustrating the positive role of potassium against the cancer are given in Section 5. This section also shows the possibility of chaotic dynamics in the discrete model. Section 6 contains discussion and conclusions.

## 2. Model formulation based on stoichiometric principles

For the theory of ecological stoichiometry, one may consult the masterpiece from Sterner and Elser [35]. According to Sui *et al*. [37], ‘ecological stoichiometry is the study of the balance of energy and multiple chemical resources (usually elements) in ecological interactions’. In this section, we formulate a mathematically tractable model that specifically deals with the dynamics of cancer-immune cell interactions in closed potassium (K ^+^)-limited environment. The above definition of ecological stoichiometry when applied to our problem can be restated as ‘cellular stoichiometry is the study of the balance of energy and multiple chemical resources (such as carbon (C) and potassium (K ^+^)) in cancer-immune cell interactions’. The greatest advantage of applying stoichiometry principles to our problem is that it allows us to employ the variability in the K ^+^ content of the cancer cell using the Droop equation for its growth.

The concept of immune-surveillance hypothesis that immune system is capable of inhibiting the growth of very small tumours and eliminating them before they become clinically evident motivates the derivation of our mathematical model of the interactions between tumour cells and immune cells. Our immune cell population may represent any of the cytotoxic immune cells (also called effector cells) such as CD8^+^ or CD4^+^ T Cells [1] of the adaptive immune system. Since adaptive immune system's effector cells proliferate in response to antigenic stimulation and kill the tumour cells, we assume that our effector cell population interacts with tumour cells in a predator–prey relationship [9,20] where immune cells play the role of the predator and the tumour cells that of prey. Without stoichiometric considerations, such a model, in a general setting, can be expressed as

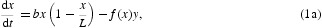

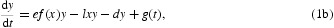

where *x* represents the size or density of the tumour cell population and *y* that of the effector cell population. The parameter *b* is the intrinsic growth rate of the tumour cell; parameter *L* represents the carrying capacity of the tumour cells; the negative term, −*f*(*x*)*y* represents the rate of killing of tumour cells by the immune cells with *f*(*x*) denoting the functional response of the immune cells; the positive term, *ef*(*x*)*y* with *e* being a positive parameter denotes the addition of immune cells in the system being activated by the presence of tumour cells; the negative term, −*lxy* represents the coercion of the immune cells by the tumour cells to their advantage; and the negative term, −*dy* depicts the apoptosis of the immune cells. The time dependent function *g*(*t*) represents the treatment term or the external antitumour activity. When *g*(*t*)=*u* (constant), the term describes continuous production of immune cells, even in the absence of cancer cells. It may be noted that we have mentioned model (1) here just for the purpose of its comparison with the model to be formulated in the following based on stoichiometric principles.

While incorporating stoichiometric reality into model (1), we concentrate on two important substances, carbon and potassium. We assume that all other substances required for proliferation of both tumour and immune cells are abundant in the system. Since the bulk of dry weight of most organisms is carbon, we express biomass of populations in carbon terms. Our model formulation approach in introducing stoichiometric considerations in model (1) follows same steps as for the KHE model in [16,37] but for suitably modifying the main assumptions of the KHE model to suit our requirements. We begin with the following assumptions:

A0. All cells are assumed to be made of carbon (C)
The total mass of the potassium *K*_t_ in the entire system is fixed; i.e. the system is closed for potassium.
Stoichiometry of the immune cells is relatively stable compared with stoichiometry of the tumour cells. Thus it is assumed, that the potassium to carbon ratio 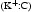 in the tumour cells varies, but it never falls below a minimum 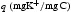; the immune cells maintain a constant 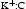 ratio, denoted by 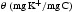.
All potassium in the system is divided into three pools: potassium in the tumour cells, potassium in the immune cells, and free potassium in the blood stream in the surrounding.

If *K*_c_, *K*_*i*_<0 and *K*_f_ denote the potassium in cancer cells, potassium in immune cells, and the free potassium in the blood stream in the surrounding, then 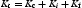. Let *Q*=*Q*(*t*) be the tumour cell's quota for K ^+^, then *K*_c_=*Qx, K*_*i*_=θ *y*. Hence

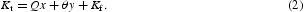

As mentioned above, denoting the tumour cell's minimal quota for potassium by *q*, the tumour's true maximal growth rate by μ_m_, its death rate by *D*, and the tumour cell's rate of killing by immune system by *f*(*x*); and using the variable-internal-stores model based on the Droop [3,4] equation that relates growth rate to the internal cell quota (also see [5,16,37]), the growth rate of the tumour cells is assumed to be governed by

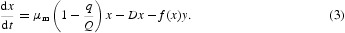

As for the dynamics of *Q* (the tumour's cell quota for K ^+^), it is assumed that *Q*’s recruitment comes proportionally from the free potassium (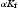, α being the proportionality constant) and it is depleted by 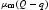 because of cell growth. This results in the following simple equation 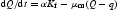. It can be verified that *Q*(*t*)≥*q* for all *t*>0 since *Q*(0)≥*q*.

Since the cell metabolic process operates in a much faster pace than the growth of total biomass of either cell species, the quasi-steady-state argument allows us to approximate *Q*(*t*) by the solution of 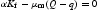. It gives

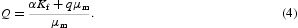

Using Equation (2), Equation (4) becomes

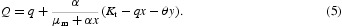

Substituting *Q* from Equation (5) into Equation (3), the equation for the growth dynamics of tumour cells can be written as

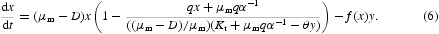

We let *e* measure the addition rate of immune cells into the system when the tumour cells are K ^+^-rich (when *Q*≥θ). If the tumour cells are K ^+^-poor (when *Q*<θ), then we assume that the addition rate suffers a reduction, and it becomes *eQ*/θ. This approach follows Liebig's [17] minimum principle and has been used in [18] model formulation. Thus, we have the following growth equation for immune cells:

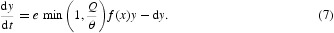

In Equation (7), parameter *d* represents the natural death rate of the immune cells.

Up till now, we considered potassium only. Now we include the possibility that carbon may also be a potentially limiting factor. It can be simply incorporated by assuming that if carbon acquisition limits the growth of the cancer cells then its population dynamics is governed by the classical logistic equation. Applying Liebig's minimum principle to potassium versus carbon limitation of the tumour cells and accordingly modifying Equation (6) and then combining it with Equation (7), we obtain the following tumour-immune cell growth model:

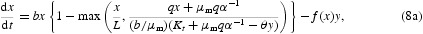

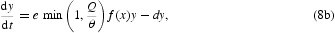

where 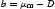 and *L* is the carrying capacity of the tumour cells.

*Remark 2.1* Model (8) is indeed the model that has been referred to and studied as the KHE model in the literature (see [16,37]) for the growth dynamics of plant and herbivore under ecological stoichiometric principles. We have simply reinterpreted it for the tumour and immune cell interactions in potassium (K ^+^)-limited environment.

We modify model (8) to consider the following model for the growth dynamics of tumour and immune cell system as our main model.

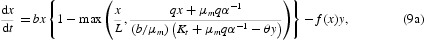

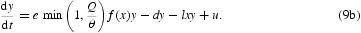

In Equation (9b), the term (−*lxy*) represents the coercing of surrounding immune cells by tumour cells into a servile role in the tumour stroma and the term (*u*) may denote a medical treatment term or any antitumour activity that may help add immune cells in the body at a constant rate.

Now the two models (1) and (9) may be compared easily. It can be seen that incorporation of stoichiometry concepts in model (1) brings in two significant changes as given below:

(a) Unlike Equation (1a) where the carrying capacity of the tumour cells is *L* (constant), the carrying capacity of the tumour cells in Equation (9a) based on stoichiometry principles depends on total potassium *K*_t_ as well as on the biomass or density of the immune cells.
(b) The production efficiency of the immune cells in Equation (1b) is considered a constant *e* whereas in Equation (9b) it depends on the ratio of the cell quota of potassium of cancer cells and immune cells.

Likewise [18], we assume that the function *f*(*x*) (in Equations (9)) that denotes the rate of killing of tumour cells by immune cells is a bounded smooth function such that

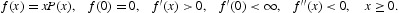

It follows from the appendix in [18] that the function *P*(*x*)=*f*(*x*)/*x* has the following properties:

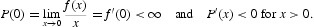

For facilitation of analysis, another version of model (9) will also be used in this paper given as

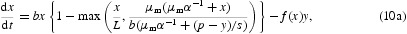

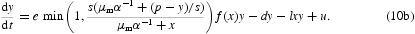

Model equations (10) can be obtained from Equations (9) by substituting 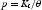, *s*=*q*/θ.

Here *p* denotes the maximal immune cells’ density allowed by the total potassium in the system and *s* is a dimensionless constant equal to the tumour cells minimal 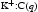 divided by the constant immune cell's 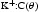. Model (10) is different from model (9) in that in Equations (10), θ is scaled out while all other parameters are retained.

The following theorem gives sufficient conditions that ensure that the solution of the model (10) (or model (9)) remains bounded.

Theorem 2.1 Let 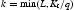. Solutions with initial conditions in the open trapezoid (or triangle if 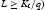


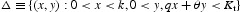

remain there for all forward times provided 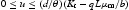.

We relegate the proof of this theorem to the appendix. This result becomes important in that it gives bounds on *u* such that if *u* is chosen within these bounds then it guarantees that the solution of model (10) (or model (9)) remains bounded. As remarked above, the term (*u*) may easily be treated as the control parameter for the tumour and directly be related to the dose of the medicine to the patient during the course of his/her treatment.

*Remark 2.2* Model (9) is a generalization of the KHE model (2.1) in [37] as it reduces to it when *l*=0 and *u*=0. The noticeable difference between the KHE model and model (9) is that they have different boundary equilibriums. While boundary equilibriums for the KHE model (or model (8) of this paper) are *E*_0_=(0, 0) and *E*_1_=(*k*, 0), model (9) has single boundary equilibrium *E*_1_=(0, *u*/*d*). For the dynamics of the KHE model and the stability of its equilibriums one may refer to various results (theorems) reported in [37].

## 3. Local stability of model (9)

To study the equilibrium solutions of model (9) and their local stability, we rewrite this model in the following form:

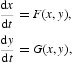

where

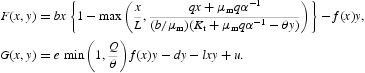



For an equilibrium solution (*x* ^+^, *y* ^+^) of model (9) satisfying the equations *F*(*x, y*)=0 and *G*(*x, y*)=0, the Jacobian matrix of the system at this equilibrium can be written as

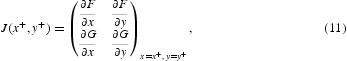

The partial derivatives of *F* and *G*, after using the notations,

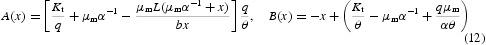

can be written as

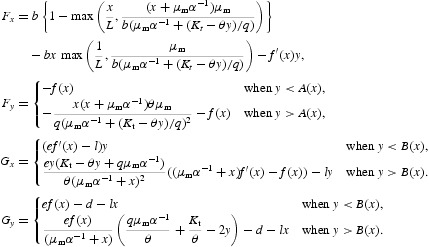



It can be seen that the model (9) has unique boundary equilibrium *E*_1_=(0, *u*/*d*). The Jacobian matrix (11) at *E*_1_ turns out to be

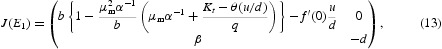

where

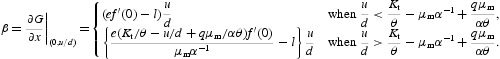



The local asymptotic stability results for the boundary equilibrium *E*_1_ can be easily obtained by studying the eigenvalues of the matrix (13). We state these results as follows:

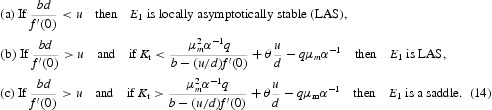

These results reveal: (i) that strengthening of immune system at larger rate may help eradicate the disease (result (a)) (ii) if the immune system is helped by the medical treatment at relatively smaller rates then while for an averaged value of *K*_t_ the disease may be eradicated (result (b)) but a large value of *K*_t_ may not eradicate the disease though in some cases (when internal equilibrium will turn out to be stable) it may help prolong the life of the patient with the disease (result (c)).

We now assume that an internal equilibrium 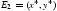 of model (9) exists. Note that −*F*_*x*_/*F*_*y*_ and −*G*_*x*_/*G*_*y*_ denote the slopes of the tumour cell and immune cell nullclines at (*x, y*) respectively. The determinant and the trace of the Jacobian matrix (11) at *E*_2_ are

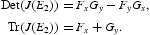

Let

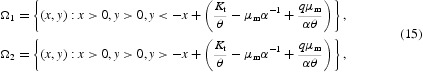

be the regions in the positive cone separated by the line 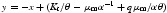. We now state and prove the (local) stability results for the internal equilibrium *E*_2_.

Theorem 3.1

(a) Let 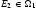  (i) Let 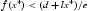. If the slope of the immune cell's nullcline at *E*_2_ is smaller than the tumour cell's (i.e. 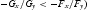, then *E*_2_ is a saddle. If the slope of the immune cell's nullcline at *E*_2_ is greater than the tumour cell's (i.e. 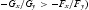 and if 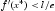 then *E*_2_ is locally asymptotically stable (LAS).
  (ii) Let 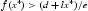. If the slope of the immune cell's nullcline at *E*_2_ is greater than the tumour cell's (i.e. 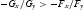) then *E*_2_ is a saddle. If the slope of the immune cell's nullcline at *E*_2_ is smaller than the tumour cell's (i.e. 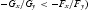 and if 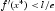 then *E*_2_ is unstable.
Let 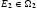  (i) Let 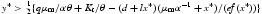. If the slope of the immune cell's nullcline at *E*_2_ is smaller than the tumour cell's (i.e. 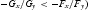, then *E*_2_ is a saddle. If the slope of the immune cell's nullcline at *E*_2_ is greater than the tumour cell's (i.e. 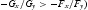 and 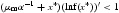 then *E*_2_ is LAS.
  (ii) Let 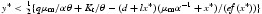. If the slope of the immune cell's nullcline at *E*_2_ is greater than the tumour cell's (i.e. 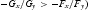 then *E*_2_ is a saddle. If the slope of the immune cell's nullcline at *E*_2_ is smaller than the tumour cell's (i.e. 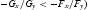 and 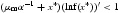 then *E*_2_ is unstable.

*Proof* Part (a)

(i) Obviously *F*_*y*_<0 and *G*_*y*_<0 at *E*_2_. Since

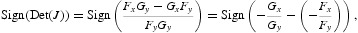

it follows that 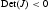 and *E*_2_ is a saddle if 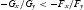. Now if 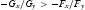 then 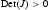. The condition 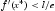 yields *G*_*x*_<0. Then 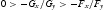 gives *F*_*x*_<0 and hence 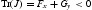. Thus *E*_2_ is LAS.
(ii) In this case, *F*_*y*_<0 and *G*_*y*_>0. Since

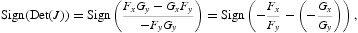

it follows that 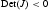 and *E*_2_ is a saddle if 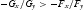. Now if 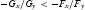, then 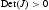. The condition 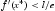 yields *G*_*x*_<0. Then 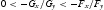 implies *F*_*x*_>0 and thus 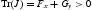. Hence *E*_2_ is unstable. Results of Part (b) can be proved similarly.

*Remark 3.1* Local stability results of Theorem 3.1 are only partial results. Results for other situations could not be discussed as in those situations signs of the partial derivatives of *F* and *G* could not be determined.

## 4. A discrete analogue of model (9)

In this section, we consider a discrete analogue of the continuous model (9). We do so for three main reasons: (i) discrete time models may be more appropriate for application in experiments where data are collected on discrete time intervals or periodically, (ii) a comparison of the results of continuous and discrete models would give better idea about the robustness of the results of the continuous model on discrete time scale and (iii) by knowing the dynamics of both versions (continuous and discrete) of the models by theoretic analysis, we can be in a better position to justify which model type would fit well to the experimental investigations. Unfortunately, there have been very few instances, for example [21], where experimental or clinical results have been compared with predictions of mathematical models. There can be several ways of deriving discrete time versions of dynamical systems corresponding to a continuous time formulation. Here we follow the method used in [8]. Assuming that the per capita growth rates in Equation (9) change only at *t*=0, 1, 2 … , then model (9) can be written as

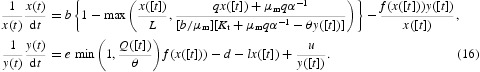

Here [*t*] denotes the integral part of 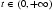. On any interval 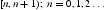, we can integrate Equation (16) and obtain the following equations for *n*≤*t*<*n*+1:

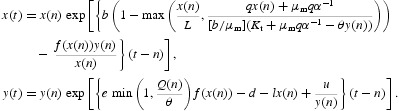

Letting *t*→*n*+1 gives

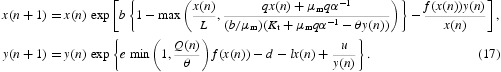

Equation (17) represent a discrete time analogue of model (9). In the following, we focus our attention on the study of equilibrium solutions of model (17) and their local asymptotic stability. To facilitate this analysis, we rewrite this model as

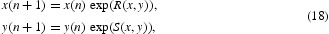

where

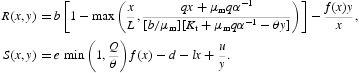



The fact that the model (9) and its discrete analogue (17) have the same equilibrium solutions implies that model (17) has unique boundary equilibrium *E*_1_=(0, *u*/*d*) and one (possibly multiple) internal equilibrium (equilibriums). We have denoted one such internal equilibrium by *E*_2_ in Section 3.

The Jacobian of Equations (18) is

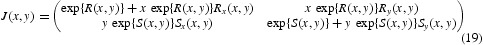

where the partial derivatives of *R* and *S* read as

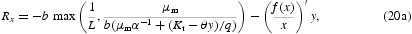

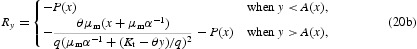

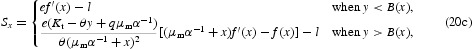

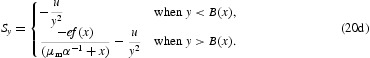

Here functions *A*(*x*) and *B*(*x*) are same as given in Equations (12).

The Jacobian matrix (19) at *E*_1_ turns out to be

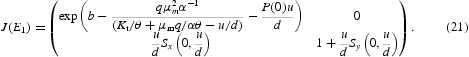

Following results can be easily verified by studying the characteristic roots of the matrix (21).

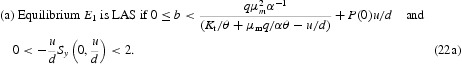

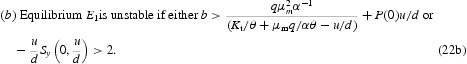

Now we discuss the local stability of internal equilibrium *E*_2_. The Jacobian matrix (19) at *E*_2_ becomes

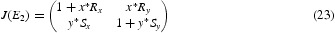

where

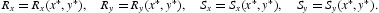

The trace and the determinant of the matrix (23) are

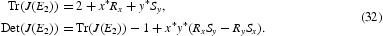

We will be using the following standard Jury test [6] to prove the local asymptotic stability results for *E*_2_.

lemma 4.1 Let A be a 2×2 constant matrix. Both characteristic roots of A have magnitude less than 1 if and only if

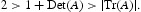

Our main local asymptotic stability results are given in the following theorem.

Theorem 4.1 If the slope of the immune cell's nullcline at *E*_2_ is smaller than the tumour cell's (i.e. 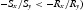, then *E*_2_ is unstable. If the slope of the immune cell's nullcline at *E*_2_ is greater than the tumour cell's (i.e. 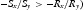 and 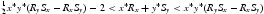, then *E*_2_ is LAS.

*Proof* In both regions Ω_1_ and Ω_2_, *R*_*y*_<0 and *S*_*y*_<0. Thus

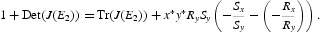



Now if 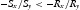 at *E*_2_, then it follows that 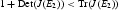 and hence the desired result of Theorem 4.1 holds true because the inequality of Lemma 4.1 is violated. On the other hand, if 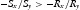 at *E*_2_, then it can be shown that whenever the inequality 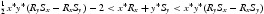 holds true then the inequality of Lemma 4.1 holds and hence the desired result of Theorem 4.1 follows.

*Remark 4.1* 

(i) It can be seen that a substitution *l*=0 and *u*=0 in the discrete model (17) leads us to the discrete KHE model (3.2) of Sui *et al*. [37]. It can also be noticed by comparing Equations (20) of this paper with Equation (3.9) of Sui *et al*. [37] that the nullcline functions *R* and *S* used in this paper retain same properties as displayed by corresponding functions of the KHE model. More specifically, the function *S*_*x*_ (*x, y*) (*G*_*x*_ (*x, y*) in [37]) changes its sign from (+ve) in region Ω_1_ to (−ve) in region Ω_2_ and the function *S*_*y*_ (*x, y*) becomes zero in region Ω_1_.
(ii) It is interesting to note that parameters *l* and *u* may produce some new situations. For example, a simple manoeuvring in *l* in Equation (20c) can change the sign of *S*_*x*_ in either region thus affecting the dynamics. Choosing suitable value of *l* and considering 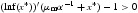 may even change the sign of *S*_*x*_ from (−ve) in region Ω_1_ to (+ve) in region Ω_2_. But we have not studied these situations in this paper.

## 5. Numerical simulations

In this section, we present some numerical simulations for the continuous model (9) and the discrete model (17). We choose the Monod type function 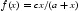 as the functional response function of the immune system. All the numerical simulations are done with MATLAB. We will consider parameter values adapted by Loladze *et al*. [18], Kuang *et al*. [16], and Sui *et al*. [37] as our reference data set. We reproduce this data set in Table 1.

**Table 1.** Reference data set for model parameters.

Parameter	Value	Unit
*K*_t_	0.025	
*e*	0.8	
*b*	1.2	day ^−1^
*d*	0.25	day ^−1^
θ	0.03	
*q*	0.0038	
*c*	0.81	day ^−1^
*a*	0.25	
μ_m_	1.2	day ^−1^
α	10	day ^−1^
*L*	0.25–2.0	

As pointed out in [37], the condition 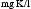 is satisfied with the initial conditions 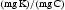 and 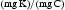 when *l*=0, *u*=0. Naturally this condition will remain satisfied when *l* and *u* are different from zero but small. We do not claim that the data of Table 1 represent any clinical situation. We have picked up these data because they provide desired negative signs of partial derivatives *G*_*x*_ and *S*_*x*_ giving stability changes for equilibriums and ensuring interesting dynamics. Moreover, same data closely mimic laboratory experiments investigating stoichiometric aspects of phytoplankton–zooplankton interactions [36,38] and thus in a sense represent biologically realistic values.

**Fig. 1.**
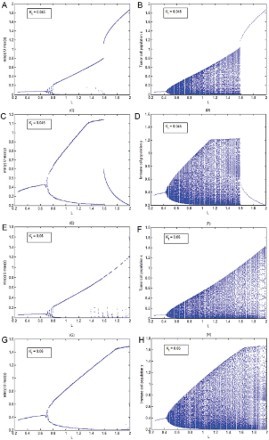
In this figure *l*=0.2, *u*=0.05 are fixed and values of rest of the parameters are chosen from Table 1. (A), (C), (E) and (G) are bifurcation diagrams of the continuous model (9) while (B), (D) (F) and (H) are corresponding bifurcation diagrams of the discrete model (17). The carrying capacity of the tumour cell population *L* is considered as bifurcation parameter.

The main purpose of this section is to numerically investigate the dynamics of both the continuous model (9) and the discrete model (17) with the variation in the total potassium in the surrounding of the tumour and see whether increasing potassium K ^+^ has any protective role against the cancer as has been suggested by Jansson studies [13,14] and very recently by Bravi *et al*. [2]. It can be seen through the bifurcation diagrams (Figure 1) that our continuous model (9) and its discrete analogue model (17) both support the protective role of potassium. In these bifurcation diagrams, the carrying capacity *L* of the tumour cells is taken as the bifurcation parameter. It is observed that when 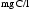, *l*=0.2, *u*=0.05 are fixed and other parameter values are chosen from Table 1, then immune system fights well against averaged size tumours but it can fail for large size tumours that threat to win ultimately (Figure 1(A)–(D)). But when the value of *K*_t_ is raised from 0.045 to 0.06 keeping all other parameters values fixed, then immune system shows strength to fight even larger size tumours. Here, the disease is not eradicated but it seems likely that the patient can live with disease all through his life (Figure 1(E)–(H)).

### 5.1. Chaotic dynamics

**Fig. 2.**
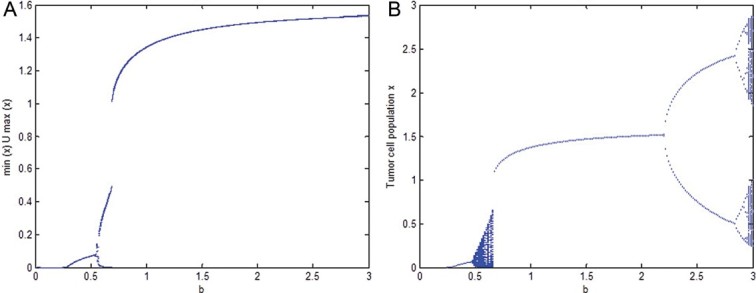
In this figure *l*=0.009, *u*=0.02, *L*=1.6 are fixed and values of rest of the parameters are chosen from Table 1. (A) is the bifurcation diagram of the continuous model (9), while (B) is the corresponding bifurcation diagram of discrete model (17). The intrinsic growth rate of the tumour cell population *b* is considered as bifurcation parameter.

In this section, we present one situation just for illustration that the dynamics of the continuous model (9) and the discrete model (17) may differ at times. The following bifurcation diagrams (Figure 2(A) and 2(B)) with *b*, the intrinsic growth rate of tumour cells, as bifurcation parameter show that the dynamics of the two models almost match for small and averaged values of *b* but for large *b*, while the dynamics of the continuous model (9) shows coexistence of tumour cells and immune cells at equilibrium values but the dynamics of the discrete model (17) exhibits chaotic dynamics with a route to chaos through periodic doubling.

## 6. Discussion and conclusions

Potassium (K ^+^) is an essential mineral found in most foods. It is a mineral that is required along with sodium and calcium for the body to work normally. It helps regulate major body functions including normal heart rhythm, blood pressure, water balance in the body, nerve impulses, muscle contractions and pH balance. The body cannot make potassium on its own and must get it from foods. Potassium is found in foods such as apricots, potatoes, bananas, oranges, pineapples, green leafy vegetables, whole grains, beans, nuts and lean meat. Most people get all the potassium they need from what they eat and drink.

The starting point for this paper has been the protective nature of potassium against the cancer risk as has been suggested by Jansson [13,14] studies. An Italian case-control study [2] recently reported significant inverse trends in pancreatic risk for increasing intake of potassium. The present paper focuses on the role of a single element, i.e. potassium on cancer etiology by developing the model using the principles of stoichiometric theory. This brings in two significant changes in the classical approach of model formulation without stoichiometric considerations. Firstly, it makes the carrying capacity of the cancer cell population dependent on the total potassium as well as the immune cell population (see Equation (9a)). Secondly, it makes the growth of immune cell population dependent on the ratio of potassium quota for cancer cell and immune cell (see Equation (9b)). Another noticeable important change that the stoichiometric theory introduces into the model (9) different from classical predator–prey relationship type formulation is the possible sign change in *G*_*x*_ (*x, y*) from positive (+ve) to negative (−ve) and vice versa (see model (9) and its representation in Section 3). The positive sign of *G*_*x*_ would denote successful immunosurveillance by the immune system while negative sign of *G*_*x*_ indicates either cancer's successful immunosuppressive activities or immune systems’ favouring approach to cancer progression. Data, supporting the dual host-protecting and tumours-sculpting actions of immunity (termed as cancer immunoediting in [39]) have been reported in the literature [29]. Vesely *et al*. [39], describe cancer immunoediting as dynamic process comprising of three distinct phases: elimination, equilibrium and escape. It is interesting to note that model (9) can produce each of immunoediting's phases for specific choices of model parameters. The elimination phase is achieved when *E*_1_=(0, *u*/*d*) is stable. Equilibrium phase is possible when 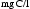 is stable. The escape phase can be attained either by having a stable equilibrium *E*_1_ (*k*, 0) under the condition *l*=0 and *u*=0 (see Remark 2.2) or by having equilibrium 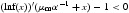 as saddle and sustenance of both cancer and immune cell populations in an oscillatory mode. The oscillatory dynamics suggested by the model (9) though is not supported by any example in solid tumours; it has been shown to occur in systemic diseases such as leukaemia [19]. It can be noted that the discrete model (17) depicts similar dynamics as mentioned above for model (9).

As pointed out earlier, the main purpose of the present paper is to investigate the protective nature of potassium against the cancer risk. It has been shown through bifurcation diagrams in Section 5 that the results of our continuous model (9) and its discrete analogue model (17) both suggest that increasing total potassium can play a protective role against cancer. It is observed that while for a small amount of potassium the immune system fights well for averaged size tumours but it may fail for larger size tumours (Figure 1(A)–(D)). On the other hand, when the amount of potassium is increased, the immune system gets stronger and it shows strength even to fight larger size tumours (Figure 1(E)–(H)). It can be noted that although Figure 1(E)–(H)) do not show the eradication of the disease but they certainly suggest a longer life for the patient. It has been noticed through numerous simulations that the continuous model (9) and the discrete model (17) mostly show similar dynamics but at times they may differ in their dynamics. A situation is illustrated in bifurcation diagram (Figure 2) when for large cancer intrinsic growth rate *b*, while model (9) shows survival of patient in cancer immunoediting equilibrium phase but model (17) suggests a chaotic dynamics having a period doubling route to chaos. Of course, the chaotic dynamics suggested by the discrete model (17) is a numerical result (a typical characteristic of discrete models) that does not have support from any experimental or clinical investigations till date but such results cannot be verified clinically or experimentally in future, who knows?

## Appendix

*Proof* [Proof of Theorem 2.1] For the proof of this theorem we use arguments similar to those in Appendices B and C of Loladze *et al*. [18]. We assume that there is time *t*_1_>0, such that a trajectory with initial conditions in Δ touches the boundary  of the trapezoid for the first time. Since the boundary consists of four sides, four cases are possible designated as *x*(*t*_1_)=0 (left border), *x*(*t*_1_)=*k* (right border), *y*(*t*_1_)=0 (bottom border) and  (top border) in [18].

Since our model differs only in equation for y from that of the model of Loladze *et al*. [18], we need to prove only that any trajectory initiated in Δ would neither cross the bottom border nor the top border. First we assume that a trajectory touches or crosses the bottom border, i.e. *y*(*t*_1_)=0, then (see, Equation (10b))

Thus it implies . This excludes the possibility that a trajectory touches or crosses the bottom border or the corners (0, 0) and (*k*, 0). Now we assume that a trajectory touches or crosses the top border i.e.

Since for all , it follows that

Substituting  from Equation (A1) into Equation (9a), it becomes

On the other hand, Equation (9b) gives,

From (A1) and (A3)

Using Equations (A4) and (A5) and the fact that *e*<1, we obtain

under the assumptions of the theorem. This contradicts Equation (A2) and hence the proof.
